# NCI10066: a Phase 1/2 study of olaparib in combination with ramucirumab in previously treated metastatic gastric and gastroesophageal junction adenocarcinoma

**DOI:** 10.1038/s41416-023-02534-1

**Published:** 2023-12-22

**Authors:** Michael Cecchini, James M. Cleary, Yu Shyr, Joseph Chao, Nataliya Uboha, May Cho, Anthony Shields, Shubham Pant, Laura Goff, Kristen Spencer, Edward Kim, Stacey Stein, Jeremy S. Kortmansky, Sandra Canosa, Jeffrey Sklar, Elizabeth M. Swisher, Marc Radke, Percy Ivy, Scott Boerner, Diane E. Durecki, Chih-Yuan Hsu, Patricia LoRusso, Jill Lacy

**Affiliations:** 1 Department of Internal Medicine (Medical Oncology), Yale University School of Medicine, New Haven, CT 06510 USA; 2 Department of Medical Oncology, Dana Farber Cancer Institute, Boston, MA 02215 USA; 3 Department of Biostatistics, Vanderbilt University, Nashville, TN 37203 USA; 4 Department of Medical Oncology & Therapeutics Research, City of Hope, Duarte, CA 91010 USA; 5 Department of Medicine, University of Wisconsin, Madison, WI 53792 USA; 6 Department of Medicine, University of California Irvine, Irvine, CA 92868 USA; 7 Karmanos Cancer Institute, Wayne State University, Detroit, MI 48201 USA; 8 Department of Gastrointestinal Medical Oncology, MD Anderson Cancer Center, Houston, TX 77030 USA; 9 Department of Medicine, Vanderbilt-Ingram Cancer Center, Nashville, TN 37203 USA; 10 Department of Medicine, Perlmutter Cancer Center of NYU Langone Health and NYU Grossman School of Medicine, New York, NY 10016 USA; 11 Department of Internal Medicine, University of California Davis, Davis, CA 95817 USA; 12 Department of Pathology, Yale University School of Medicine, New Haven, CT 06510 USA; 13 Department of Obstetrics and Gynecology, University of Washington, Seattle, WA 98195 USA; 14 Cancer Therapy Evaluation Program, National Cancer Institute, Bethesda, MD 20892 USA

**Keywords:** Gastric cancer, Targeted therapies

## Abstract

**Background:**

Our preclinical work revealed tumour hypoxia induces homologous recombination deficiency (HRD), increasing sensitivity to Poly (ADP-ribose) polymerase inhibitors. We aimed to induce tumour hypoxia with ramucirumab thereby sensitising tumours to olaparib.

**Patients and methods:**

This multi-institution single-arm Phase 1/2 trial enrolled patients with metastatic gastroesophageal adenocarcinoma refractory to ≥1 systemic treatment. In dose escalation, olaparib was evaluated at escalating dose levels with ramucirumab 8 mg/kg day 1 in 14-day cycles. The primary endpoint of Phase 1 was the recommended Phase 2 dose (RP2D), and in Phase 2 the primary endpoint was the overall response rate (ORR).

**Results:**

Fifty-one patients received ramucirumab and olaparib. The RP2D was olaparib 300 mg twice daily with ramucirumab 8 mg/kg. In evaluable patients at the RP2D the ORR was 6/43 (14%) (95% CI 4.7–25.6). The median progression-free survival (PFS) was 2.8 months (95% CI 2.3–4.2) and median overall survival (OS) was 7.3 months (95% CI 5.7–13.0). Non-statistically significant improvements in PFS and OS were observed for patients with tumours with mutations in HRD genes.

**Conclusions:**

Olaparib and ramucirumab is well-tolerated with efficacy that exceeds historical controls with ramucirumab single agent for gastric cancer in a heavily pre-treated patient population.

## Introduction

Gastric cancer remains one of the largest global causes of cancer-related death, and while the incidence has declined in the United States, the incidence of the lower oesophageal and gastroesophageal junction (GEJ) adenocarcinoma has increased dramatically over the past several decades in western countries [1]. Thus, new treatments are required to address this unmet need. The National Comprehensive Cancer Network guidelines for metastatic gastroesophageal adenocarcinoma recommend initial therapy with 5-fluorouracil (5-FU)/platinum doublet in combination with an immune checkpoint inhibitor for most patients [2]. After progression on initial therapy median survival is less than 1 year, and approved second-line treatments include ramucirumab as a single agent or in combination with paclitaxel [3, 4].

Ramucirumab is a monoclonal antibody against the vascular endothelial growth factor receptor 2 (VEGFR2). By binding to VEGFR2, ramucirumab prevents VEGF ligands from binding to VEGFR2 and ultimately impairs tumour-related angiogenesis. Substantial preclinical work has shown that hypoxia can induce homologous recombination deficiency (HRD) in tumours. Hypoxia reduces the expression of *BRCA1* and *RAD51* through promoter modification by E2FR complexes [5, 6]. Furthermore, hypoxia has also been shown to directly impair homologous recombination and leads to increased sensitivity to inhibitors of poly (ADP-ribose) polymerase (PARP) in preclinical models [7, 8]. These principles have been translated into the clinic with the combination of cediranib and olaparib as well as maintenance olaparib and bevacizumab [9–12]. Thus, ongoing clinical investigation of PARP inhibitors with anti-tumoral agents targeting angiogenesis is warranted.

Genomic signatures suggest that up to 12% of gastric cancer has evidence of HRD at baseline [13]. Furthermore, loss of expression in the DNA repair gene ataxia telangiectasia mutated (ATM) is reported in up to 22% of tumours [14, 15]. These observations ultimately led to the evaluation of olaparib plus paclitaxel in the GOLD clinical trial, compared to single-agent paclitaxel, which did demonstrate improved progression-free survival (PFS), but not overall survival (OS) benefit over single-agent paclitaxel [16, 17]. While paclitaxel is an active agent for gastroesophageal cancer, the overlapping toxicities with olaparib and lack of significant synergy with olaparib may partially explain the negative results. Taken together, these observations suggest that future PARP inhibitor strategies for gastric cancer should focus on synergistic combinations that do not have overlapping toxicity, many of which are chemotherapy-free.

Given the supporting preclinical data that hypoxia sensitises tumours to PARP inhibitors, we proposed to evaluate the combination of a PARP inhibitor in combination with ramucirumab, an approved treatment targeting angiogenesis in metastatic gastric and GEJ adenocarcinoma. Due to non-overlapping toxicity profiles, we hypothesised that olaparib and ramucirumab would be well-tolerated and that ramucirumab would sensitise tumours to olaparib, thus enhancing overall response rates (ORR) compared to historical control of 3% with single-agent ramucirumab [3]. Here, we report the safety and efficacy of the combination of olaparib and ramucirumab in metastatic gastric and GEJ adenocarcinoma evaluated in NCI10066: a Phase 1/2 open-label multicenter centre clinical trial through the National Cancer Institute Experimental Therapeutics Clinical Trials Network (ETCTN).

## Methods

### Study design and participants

NCI10066 was a single-arm open-label Phase 1/2 clinical trial open through the National Cancer Institute (NCI) ETCTN. Eligible patients had stage IV, histopathologically confirmed gastric or GEJ adenocarcinoma that had progressed after at least one standard therapy in the metastatic setting. The study consisted of a dose escalation portion followed by dose expansion. The dose escalation proceeded with a standard 3 + 3 design with a dose-limiting toxicity evaluation period for 28 days from treatment initiation. In dose expansion, enrolment proceeded at the RP2D. Eligible patients were 18 years of age or older, had measurable disease according to Response Evaluation Criteria in Solid Tumours (RECIST, version 1.1), had an Eastern Cooperative Oncology Group (ECOG) performance score of 0 or 1, and had adequate organ function (as assessed by renal, hepatic, and haematologic parameters). Patients were excluded if they had previously received ramucirumab. The NCI Cancer Therapy Evaluation Program and the NCI Central Institutional Review Board approved the protocol and all patients provided written informed consent. The full study protocol is available in the supplementary data. The study was conducted in accordance with the Declaration of Helsinki and followed the Consolidated Standards of Reporting Trials. Trial registration: ClinicalTrials.gov identifier: (NCT03008278).

### Procedures

In the Phase 1 dose escalation, participants received escalating doses of olaparib twice daily with ramucirumab 8 mg/kg on day 1 in 14-day cycles. Two dose levels of olaparib 200 (dose level 1) and 300 mg twice daily (dose level 2) were evaluated in a 3 + 3 design. In the Phase 2 dose expansion, participants received the RP2D of olaparib with the standard dose of ramucirumab at 8 mg/kg every 14 days. Dose modifications were allowed to manage clinically significant toxicities as specified in the protocol. Participants received study treatment until radiographic or symptomatic progression, unacceptable toxicity, death, or withdrawal from the study. Radiographic tumour assessments were performed at baseline and every 6 weeks for the duration of the study.

Molecular profiling was performed on formalin-fixed paraffin-embedded tissue from pre-treatment biopsies by whole exome sequencing (WES) and targeted sequencing with the BROCA-HR panel. An on-treatment biopsy was changed from mandatory to optional in a protocol amendment prior to any patients enrolling to facilitate enrollment. The WES was performed on the Ion Torrent sequencing platform, utilising IonAmpliSeq Exome RDY library kits with 60 ng of genomic tumour DNA extracted from formalin-fixed paraffin-embedded tissue sections and normal tissue from the same patient. Templating of libraries on chips was carried out using an Ion CHEF instrument and sequencing done on an S5XL instrument, followed by analysis with Torrent Suite software. The BROCA-HR panel was run on normal and tumour tissue using the Illumina platform covering 73 genes including clinically relevant HRD genes, and was performed at the University of Washington (a full list of the BROCA-HR panel is available in the Appendix). As pre-specified in the protocol a tumour was considered to have HRD if an inherited or somatic pathogenic variant was present in any of the following 17 HRD genes and non-HRD in the absence of these mutations: *ATM, ATR, BARD1, BLM, BRCA1, BRCA2, BRIP1, CHEK2, MRE11A, NBN, PALB2, RAD51B, RAD51C, RAD51D, RBBP8, SLX4, XRCC2, CDK12*. Results for these 17 genes are available using the NCI-validated BROCA-HR panel. These 17 genes were selected based on available evidence at the time of clinical trial development by the investigators and NCI, which were thought to confer sensitivity to PARPi based on evidence from the BROCA-HR panel validation for HRD in ovarian cancer [18, 19].

### Outcomes

The primary endpoint in Phase 1 dose escalation was the determination of the RP2D. In the Phase 2 dose expansion, the primary endpoint was the ORR, defined as the proportion of patients with either a complete or partial response by RECIST (version 1.1) [20]. Tumour profiling with WES or BROCA-HR was used to assess for mutations in the 17 pre-specified HRD genes as an integrated biomarker to stratify the ORR results. Based on a historical control for ramucirumab as single agent, the null hypothesis was ≤5% ORR and an alternative hypothesis of 25% in patients with HRD mutations and 20% without. Secondary endpoints included PFS, OS, BROCA-HR status, and incidence of adverse events.

### Statistical analysis

Descriptive statistics, including medians and interquartile ranges for continuous variables, as well as percentages and frequencies for categorical variables. The overall response rate with the 95% confidence interval (CI) was calculated by the Clopper-Pearson method. The therapy would be worthy of further investigation if there were ≥1 response in tumours with HRD alterations and ≥4 responses in tumours without evidence of HRD alterations, where the log-rank test was used. The PFS and OS rates with the 95% CIs were estimated using the Kaplan–Meier method. The medians of PFS and OS with the 95% CIs were estimated by the Brookmeyer–Crowley method. Moreover, the median follow-up time was estimated by the reverse Kaplan–Meier method [21]. All analyses mentioned above were performed using R 4.2.3, and the R packages survival 3.2-7 and survminer 0.4.9.

## Results

**Fig. 1:**
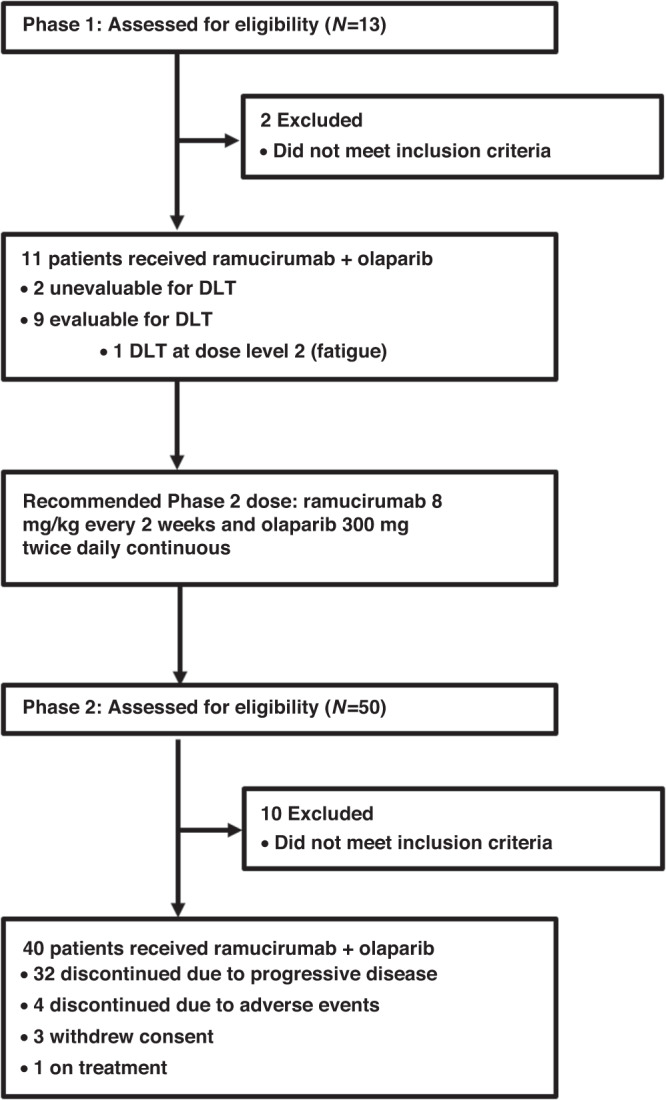
Trial profile. Flow chart for enrollment indicating patient course for phase 1 and phase 2.

Between 2/8/18 and 9/17/21, 51 patients were enrolled in NCI10066 at ten centres throughout the ETCTN (Fig. 1). In Phase 1 dose escalation, 11 patients received study treatment, and 40 patients received treatment in Phase 2 dose expansion. The baseline characteristics for all enrolled patients are listed in Table 1. All patients had previously received at least one prior line of therapy and 28/51 (55%) had received at least two prior lines of therapy. Furthermore, all patients received prior platinum combination chemotherapy, but platinum sensitivity was not available in the case report forms. At the time of the April 22, 2022 data cutoff, all patients had discontinued study treatment.

**Table 1:** Baseline characteristics of the intention-to-treat population.

Characteristic	Ramucirumab and olaparib (*N* = 51)
Age	
Median	64
Range	38–82
Sex—no. (%)	
Male	40 (78)
Female	11 (22)
Race—no. (%)	
White	42 (82)
Black	1 (2)
Asian	1 (2)
American Indian or Alaska Native	1 (2)
Not reported	6 (12)
ECOG performance status—no. (%)	
0	24 (47)
1	27 (53)
Location of primary tumour—no. (%)	
Gastroesophageal Junction	31 (76)
Stomach	10 (24)
Previous Surgical Resection—no. (%)	
Yes	9 (18)
No	42 (82)
HER2 positive—no. (%)	13 (25)
Histology—no. (%)	
Well-differentiated	3 (6)
Moderately differentiated	13 (25)
Poorly differentiated	30 (59)
Not reported	5 (10)
Number of prior therapies—no. (%)	
1	23 (45)
2	13 (25)
3	11 (22)
≥4	4 (8)
Prior treatment exposure	
5-Fluorouracil	51 (100)
Platinum	51 (100)
Taxane	15 (29)
Irinotecan	10 (20)
Anti-PD-1/PD-L1	11 (22)
HER2-targeted treatment	12 (24)
Gemcitabine	1 (2)
TAS-102	2 (4)

In Phase 1 dose escalation, three patients were treated at dose level 1 and eight patients at dose level 2. All patients at dose level 1 were evaluable for DLT and there were no DLTs observed. At dose level 2, there were two patients that were not evaluable for DLT based on insufficient olaparib dosing in the 28-day DLT period. Of the six evaluable patients treated at dose level 2, there was one DLT for grade 3 fatigue. Thus, dose level 2 of olaparib 300 mg twice daily with ramucirumab 8 mg/kg on day 1 every 14 days was determined to be the RP2D. The study then proceeded to dose expansion, where 40 additional patients were treated with olaparib 300 mg twice daily with ramucirumab 8 mg/kg on day 1 every 14 days.

**Fig. 2:**
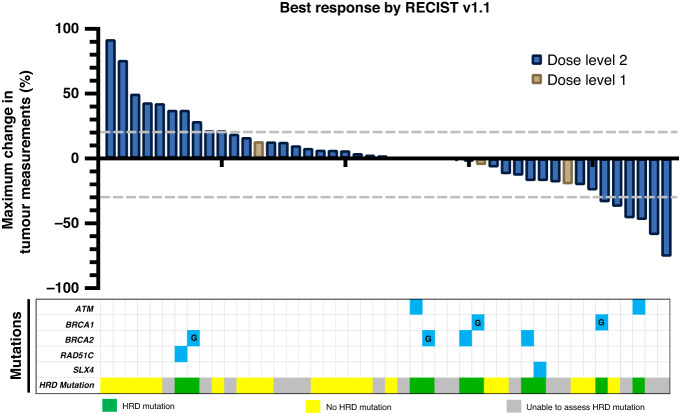
Waterfall plot for best response by RECIST version 1.1. Genomic results labelled with “G” represent germline alterations.

**Fig. 3:**
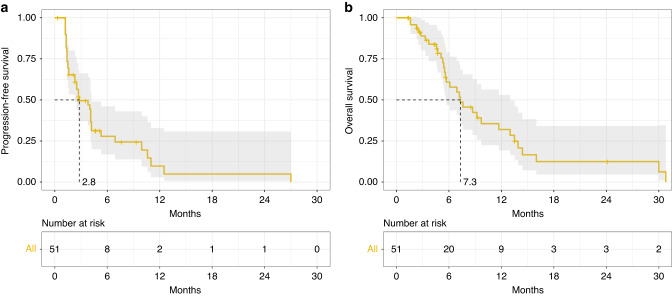
Progression-free survival (**a**, left panel) and overall survival (**b**, right panel) curves for intention-to-treat population.

**Fig. 4:**
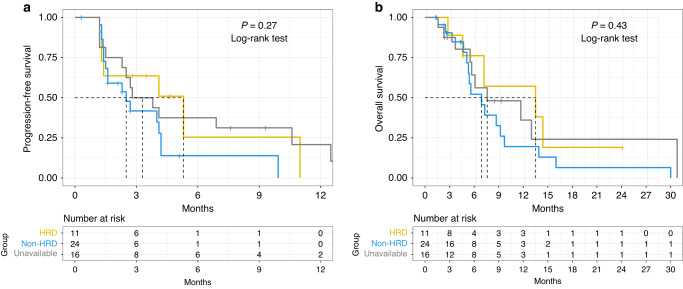
Progression-free survival (**a**, left panel) and overall survival (**b**, right panel) for patients stratified by tumour HRD status. The gold curve represents tumours with HRD, the blue curve represents tumours without HRD, and the grey represents tumours with unknown HRD status.

In the intention-to-treat (ITT) population treated at the RP2D there were 6 PRs observed in 43 evaluable patients by RECIST with an ORR of 6/43 (14%) (95% CI 4.7–25.6) (Fig. 2) and a median duration of response of 10 months (range 5–27 months). All patients that had a PR were treated at the RP2D. For patients receiving study treatment at the RP2D as second-line therapy the ORR was 4/22 (18%), and one patient with an objective response had received prior anti-PD-1 therapy. The ITT 16-week and 24-week disease control rates (partial response + stable disease) were 18/46 (40%) and 8/46 (17%), respectively. For all enrolled patients the median PFS was 2.8 months (95% CI 2.3–4.2), and median OS was 7.3 months (95% CI 5.6–13.0) (Fig. 3a, b). Genomic analysis was available for 35 patients to determine HRD status as defined by our pre-specified genomic criteria for the 17 HRD genes hypothesised at the time of protocol development to confer PARPi sensitivity. For the six patients with PRs, two had HRD-positive tumours, one HRD-negative cancer, and three did not have genomic results. When stratifying by HRD status median PFS was 5.3 months for patients with HRD-positive tumours, 2.5 months for HRD-negative tumours, and 3.3 months for HRD status unknown (*P* = 0.27). Furthermore, the median OS for HRD-positive tumours was 13.5 months, 6.9 months for HRD-negative and 7.6 months for HRD status unknown (*P* = 0.43) (Fig. 4). Additional detail for the specific genomic results is provided in Supplementary Table 1.

The median number of cycles of ramucirumab and olaparib was 5 (range 1–43), and 14 (27%) of patients required a dose reduction of olaparib. The most frequently reported treatment-related adverse events (TRAE) at any grade that occurred in more than 10% of patients are outlined in Table 2. As reported, 48 (94%) reported at least 1 TRAE, and 3 patients experienced TRAEs that resulted in discontinuation of study treatment. There were no treatment-related deaths.

**Table 2:** Frequency of treatment-related adverse events and laboratory abnormalities with >10% incidence.

Event	Any grade	Grade ≥ 3
Any event—no. (%)	48 (94)	15 (29)
Any cause serious event—no. (%)	15 (29)	15 (29)
Most common events—no. (%)		
Fatigue	32 (63)	2 (4)
Nausea	30 (59)	5 (10)
Hypertension	16 (31)	5 (10)
Vomiting	16 (31)	3 (6)
Anorexia	13 (25)	0
Headache	12 (24)	0
Diarrhoea	11 (22)	0
Constipation	10 (20)	0
Weight loss	7 (14)	1 (2)
Dysgeusia	6 (12)	0
Myalgia	5 (10)	0
Laboratory abnormalities—no. (%)		
Platelet count decreased	17 (33)	0
Anaemia	14 (27)	1 (2)
Proteinuria	14 (27)	0
Lymphocyte count decreased	8 (16)	1 (2)
Neutrophil count decreased	8 (16)	0
Hypoalbuminemia	6 (12)	0
Creatinine increased	5 (10)	0

## Discussion

The treatment landscape for metastatic gastric and GEJ adenocarcinoma has changed significantly over the past several years with the approval of immunotherapy in combination with 5-fluorouracil and platinum as initial treatment. After progression on initial therapy, approved second-line regimens are ramucirumab as monotherapy or in combination with paclitaxel. However, neuropathy from first-line treatment may complicate the use of paclitaxel as second-line treatment. Aside from ramucirumab monotherapy, there are no available second-line treatment options for gastric and GEJ adenocarcinoma that might spare patients from the toxicities of cytotoxic chemotherapy. Thus, the development of novel targeted treatment options remains an unmet need for gastric and GEJ adenocarcinoma. Here we report the results for the first study evaluating a PARP inhibitor in combination with a VEGFR2-targeted antibody in any solid tumour.

In the Phase 1 dose escalation, there were no unexpected safety signals, and the RP2D was the protocol-specified highest dose: olaparib 300 mg twice daily with ramucirumab 8 mg/kg on day 1 in 14-day cycles, which is consistent with the full dose monotherapy dose intensity for both agents. In dose expansion, 40 additional patients were enroled at the RP2D. The ORR for all enrolled patients at the RP2D evaluable for response by RECIST was 6/43 (14%), and in the intention-to-treat population the median PFS and median OS were 2.8 months and 7.3 months, respectively. These efficacy results compare favourably to ramucirumab monotherapy as the second-line Phase 3 REGARD clinical trial had a ORR of 3%, median PFS 2.1 months and median OS 5.2 months [3]. Moreover, patients in NCI10066 were more heavily pre-treated with 55% of patients receiving at least 2 prior lines of therapy and in the second-line population the ORR was 4/23 (17%), which is improved compared to historical control. Unfortunately, three patients with objective responses did not have genomic results for their tumours, and this significantly impacts our ability to interpret the efficacy of the regimen by HRD status. Furthermore, the comparison of our results to paclitaxel and ramucirumab in the RAINBOW trial is difficult because RAINBOW was entirely a second-line patient population [4]. However, based on our results it is unlikely that olaparib and ramucirumab would be superior to paclitaxel and ramucirumab in a randomised clinical trial and a future comparison to single-agent ramucirumab is more feasible. Ramucirumab monotherapy is largely a cytostatic agent and the ORR observed in our study suggests the combination may be more active than single-agent ramucirumab and warrants further study in a randomised trial. As ramucirumab was initially studied as a second-line treatment for gastric cancer, a randomised evaluation could be considered of ramucirumab and olaparib compared to ramucirumab monotherapy for patients’ ineligible for cytotoxic chemotherapy.

The genomic analysis was available for 35 patients (10 WES and 25 BROCA-HR). Eleven cancers were determined to be HRD-positive due to the presence at least one mutation in the 17 HRD genes that were pre-specified to confer PARP inhibitor sensitivity as outlined in the methods. This included 4 *BRCA2* mutations, 3 *ATM*, 2 *BRCA1*, 1 *RAD51* and 1 *SLX4* with no HRD reversion mutations detected. Two patients with RECIST v1.1 PRs had HRD-positive cancers (*ATM* and *BRCA1*, which were both biallelic alterations), while 1 patient with a PR had HRD-negative cancer, and 3 were unfortunately unable to be assessed for genomic alterations. For the 11 patients with HRD tumours, 2 patients had biallelic mutations (*ATM* and *BRCA1* – both with PR), 7 patients had tumours with monoallelic HRD mutations, and 2 were unable to be determined. Furthermore, 1 patient with a germline *BRCA1* mutation also had co-occurring biallelic *TP53B1* mutations (Supplementary Table 1), which may confer PARPi resistance by restoring homologous recombination, however, this patient achieved a PR [22–25]. There were no other co-occurring mutations or HRD reversion mutations identified that would be suspected to have an impact on treatment response. When stratifying PFS and OS by HRD status, we observed a trend towards improved PFS and OS that did not reach statistical significance, although the analysis was underpowered for these endpoints. In this heavily pre-treated patient population, the HRD-negative group PFS and OS was still higher than described in the historical control with REGARD although it is possible that HRD-positive tumours have a more favourable biology, resulting in an improved prognosis regardless of treatment. In the ITT population, 18/46 (40%) patients had ≥16-week disease control and 5/18 (27%) were HRD-positive, 6/18 (33%) were HRD-negative, and 7/18 (39%) did not have molecular results. Collectively, the efficacy data stratified by HRD status suggests that olaparib with ramucirumab may be effective in an unselected patient population, but efficacy may be enriched by pre-selecting patients with tumours that have HRD alterations. The efficacy data for ORR, PFS, and OS is summarised by HRD status in the swimmer’s plot (Supplementary Fig. 1).

The NCI10066 study is limited due to the sample size and lack of randomisation which makes it difficult to generalise these findings to all patients with gastric cancer. The patient heterogeneity in regard to number of prior treatments also complicates the interpretation of the efficacy endpoints, and may ultimately result in an underestimation of the ORR and survival of the combination. While the genomic results are available for many patients, given the mechanism of olaparib, a more comprehensive genomic analysis of the study population would strengthen the conclusion that the olaparib and ramucirumab combination works regardless of HRD. Furthermore, while our definition of HRD for data analysis was determined at the time of study development, a more modern definition of HRD genes and functional HRD scores that also accounts for platinum sensitivity would be more appropriate for a future clinical trial. Ultimately a randomised study in a more uniform patient population will be needed to make more definitive measurements for the efficacy of the treatment combination. Other future development strategies for olaparib and ramucirumab may be in combination with immune checkpoint inhibitors given the immunogenicity of gastroesophageal cancer, non-overlapping toxicity profiles, and potential for synergy in preclinical models [26–32].

In conclusion, the NCI10066 trial confirmed the safety of olaparib 300 mg twice daily with ramucirumab 8 mg/kg on day 1 in 14-day cycles. The ORR, PFS, and OS with olaparib and ramucirumab are all superior compared to the historical control of ramucirumab monotherapy for metastatic gastric cancer. Future clinical trials could include ramucirumab and olaparib compared to ramucirumab monotherapy as a chemotherapy-free approach for patients’ ineligible for cytotoxic chemotherapy. In NCI10066, we observed anti-tumour activity for patients with and without tumour HRD, which suggests the combination of ramucirumab and olaparib may be superior to ramucirumab monotherapy and a randomised trial is warranted for gastric cancer in the second-line treatment setting.

### Supplementary information

supplementary figure 1

Supplementary Sequencing Table

## Data Availability

The genomic data for the BROCA-HR panel are publicly available in Sequence Read Archive BioProject ID PRJNA1037163. Additional data may be made available based on reasonable request to the corresponding author.
