# Supplementary material for: NCI10066: a Phase 1/2 study of olaparib in combination with ramucirumab in previously treated metastatic gastric and gastroesophageal junction adenocarcinoma
Source: Br J Cancer. 2023 Dec 22;130(3):476–82. doi: 10.1038/s41416-023-02534-1 (PMC10844282; doi:10.1038/s41416-023-02534-1)
Supplement: Supplementary file 1 — supplementary figure 1 [file 41416_2023_2534_MOESM1_ESM.pdf]

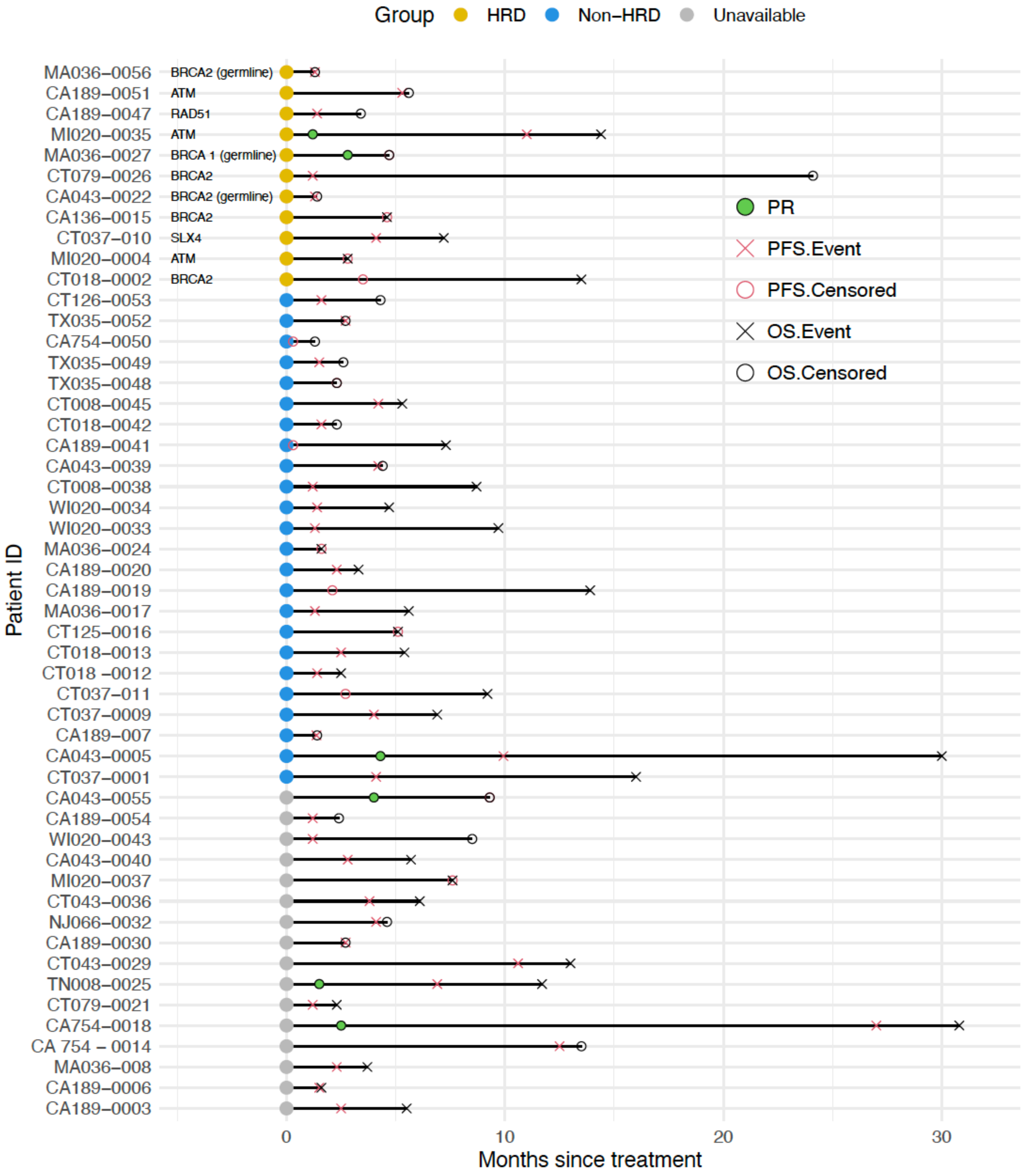

Supplementary Figure 1. Swimmer's plot for all enrolled patients showing timing of objective response in relation to duration of treatment.
