# Supplementary material for: NCI10066: a Phase 1/2 study of olaparib in combination with ramucirumab in previously treated metastatic gastric and gastroesophageal junction adenocarcinoma
Source: Br J Cancer. 2023 Dec 22;130(3):476–82. doi: 10.1038/s41416-023-02534-1 (PMC10844282; doi:10.1038/s41416-023-02534-1)
Supplement: Supplementary file 2 — Supplementary Sequencing Table [file 41416_2023_2534_MOESM2_ESM.docx]

Supplementary Table 1. Detailed sequencing results for patients with molecular results.

| **ID** | **Panel** | **Gene** | **Position** | **Transcript Code Change** |
| --- | --- | --- | --- | --- |
| 1 | WES | MUC6  TP53 | chr11:1016255  chr17:7578475 | c.6543_6545delCTC  c.455C>T |
| 2 | BROCA-HR | SMARCA4  CDKN2A  TP53  ERBB2  BRCA1*  PARP1  TP53  ERBB2  MYC  ABCB1 | chr19:11097070  chr9:21974696  chr17:7577121  Unidentified breakpoint  chr17:41209079-41209080  chr1:226550815  chr17:7578475  Unidentified breakpoint  Unidentified breakpoint  Unidentified breakpoint | c.561C>A  c.130_131insG  c.817C>T  Whole Gene Amplification  c.5266dup  c.2833A>C  c.455C>T  Whole Gene Amplification  Whole Gene Amplification  Whole Gene Amplification |
| 3 | WES | ATM  CTNNB1  PIK3CA ERBB3  STK11 | chr11:108098585  chr3:41266137  chr3:178952085  chr12:56482341  chr19:1220395 | c.155G>T  c.134C>T  c.3140A>G  c.889G>T  c.493_501delGAGTACCTG |
| 4 | WES | CTNNB1  FLT3  GNAS  MAP2K1  NFE2L2  PIK3CA PIK3R1  TP53 | chr3:41266137  chr13:28623625  chr20:57484420  chr15:66729175  chr2:178098944  chr3:178916876  chr5:67522532  chr17:7578406 | c.134C>T  c.932G>A  c.2530C>T  c.383G>T  c.101G>A  c.263G>A  c.29C>T  c.524G>A |
| 5 | BROCA-HR | CDKN2A  TP53 | chr9:21971080  chr17:7578454 | c.278C>G  c.476C>A |
| 6 | BROCA-HR | SMARCA4  TP53  CDKN2A | chr19:11134269  chr17:7577580  chr9:21974692-21974721 | c.2935C>T  c.701A>G  c.106_135del |
| 7 | WES | SLX4  MUC6  TP53 | p.Arg408Trp  p.Pro2341Leu  p.Gln165Ter | c.1222C>T  c.7022C>T  c.493C>T |
| 8 | BROCA-HR | KRAS  CCND1  CDKN2A: RBFOX3 | chr12:25398284  Unidentified breakpoints  chr9:21974488(-)::chr1777184795(+) | c.35G>T  Whole gene amplification  NM_000077 (CDKN2A) intron 1 (+) :: NM_001350451 (RBFOX3) intron 3 (-) |
| 9 | WES | TP53 | chr17:7578272 | c.577C>A |
| 10 | WES | PIK3CA  TP53 | chr3:178936082  chr17:7577120 | c.1624G>A  c.818G>A |
| 11 | WES | ARID1A  CDKN2A  BRCA2  TP53 | chr1:27106861  chr9:21971108  chr13:32913213  chr17:7577539 | c.6472C>T  c.250G>A  c.4721A>C  c.742C>T |
| 12 | WES | None | N/A | N/A |
| 13 | WES | TP53 | chr17:7579329 | c.358A>G |
| 14 | BROCA-HR | PARP1  TP53  ERBB2  MYC  ABCB1 | chr1:226550815  chr17:7578475  Unidentified breakpoints  Unidentified breakpoints  Unidentified breakpoints | c.2833A>C  c.455C>T  Whole gene amplification  Whole gene amplification  Whole gene amplification |
| 15 | BROCA-HR | TP53  ARID1A CDKN2A | chr17:7577097  chr1:27023855  chr9:21968160-22246675 | c.841G>T  c.961dupC  Whole gene deletion |
| 16 | BROCA-HR | BRCA2* | chr13:32914068-32914071 | c.5576_5579del |
| 17 | WES | TP53  SMARCA4 | chr17:7577580  chr19:11134269 | c.701A>G  c.2935C>T |
| 18 | BROCA-HR | CDKN2A  TP53  BRCA2 | chr9:21974751  chr17:7578403  chr13:32915496(+)::chr13:33578228(-) | c.76G>T  c.527G>A  Translocation |
| 19 | BROCA-HR | NF1  PTEN  SMARCA4  TBP53BP1  TP53BP1  RPL22  BRCA1* | chr17:29562684  chr10:89653797  chr19:11132536  chr15:43701236  chr15:43749179  chr1:6253117-6253119  chr17:41243838 | c.3764delA  c.95T>G  c.2752C>T  c.5459A>G  c.1627G>T  c.120-5_120-3delinsCAG  c.3710delT |
| 20 | BROCA-HR | TP53  NRAS MYC | chr17:7578213  chr17:7578213  Unidentified breakpoints | c.636delT  c.182A>G  Whole gene amplification |
| 21 | BROCA-HR | TP53  CCND1 | chr17:7574003  Unidentified breakpoints | c.1024C>T  Whole gene amplification |
| 22 | BROCA-HR | ATM  SMARCA4  TP53 | chr11:108198397  chr19:11141426  chr17:7577548 | chr17:7577548  c.3403C>T  c.733G>A |
| 23 | BROCA-HR | MSH6* | chr2:48028094 | c.2972C>T |
| 24 | BROCA-HR | RB1  TP53  KRAS | chr13:48951087-48951088  chr17:7577014-7577017  Unidentified breakpoints | c.1249_1250del  c.919+2_919+5delinsA  Whole gene amplification |
| 25 | BROCA-HR | MYC  CCND1  ERBB2  TP53  TSC2 | Unidentified breakpoints  Unidentified breakpoints  Unidentified breakpoints  chr17:7590693-7590724  chr17:7590693-7590724 | Whole gene amplification  Whole gene amplification  Whole gene amplification  c.-58_-29+2del  c.5398G>A |
| 26 | BROCA-HR | TP53  CDKN2A | chr17:7577556  chr9:21971111 | c.725G>A  c.247C>T |
| 27 | BROCA-HR | TP53  CCND1  MYCN  ERBB2 | chr17:7577574  Unidentified breakpoints  Unidentified breakpoints  Unidentified breakpoints | c.707A>G  Whole gene amplification  Whole gene amplification  Whole gene amplification |
| 28 | BROCA-HR | FBXW7  TP53  RAD51  ERBB2  CDK12  MYC  CCNE1 | chr4:153249388  chr17:7577106  chr15:41021702  Unidentified breakpoints  Unidentified breakpoints  Unidentified breakpoints  Unidentified breakpoints | c.1390G>A  c.832C>T  c.648-1G>A  Whole gene amplification  Whole gene amplification  Whole gene amplification  Whole gene amplification |
| 29 | BROCA-HR | TP53  PIK3CA  KRAS  CCND1 | chr17:7578406  chr3:178948134  Unidentified breakpoints  Unidentified breakpoints | c.524G>A  c.2906A>G  Whole gene amplification  Whole gene amplification |
| 30 | BROCA-HR | TP53  MYC  CCND1  MYCN  ERBB2  EPCAM* | chr17:7578403  Unidentified breakpoints  Unidentified breakpoints  Unidentified breakpoints  Unidentified breakpoints  chr2:47606078 | c.527G>A  Whole gene amplification  Whole gene amplification  Whole gene amplification  Whole gene amplification  c.556-14A>G |
| 31 | BROCA-HR | TP53  CCNE1  ERBB2  CCND1 | chr17:7579377  Unidentified breakpoints  Unidentified breakpoints  Unidentified breakpoints | c.310C>T  Whole gene amplification  Whole gene amplification  Whole gene amplification |
| 32 | BROCA-HR | RB1  ERBB2  POLE3  TP53  ATM | chr13:48916769  chr17:37881396  chr12:133252325  chr17:7578226  chr11:108098354 | c.299G>A  c.2588A>G  c.2588A>G  c.623A>T  c.3G>T |
| 33 | BROCA-HR | TP53  TSC1  RAD50  ABCB1  MYC | chr17:7578406  chr9:135778166  chr5:131930560  Unidentified breakpoints  Unidentified breakpoints | c.524G>A  c.2217G>T  c.1794-1G>T  Whole gene amplification  Whole gene amplification |
| 34 | BROCA-HR | TP53  EZH2  CHD4 | chr5:131930560  Unidentified breakpoints  chr12:6696681 | c.818G>A  exon 2-8 del  c.3748G>A |
| 35 | BROCA-HR | BRCA2* | chr13:32968951 | c.9382C>T |

The sequence position is aligned to the genome version GRCh37/gh19

*Denotes germline variant
